# Medical Extracts

**Published:** 1885-12

**Authors:** 


### Medical Extracts.

### A Case of Impacted Rhinolith.

Chiari relates the details of the case of a lady who was sent to him by Nothnagel, and who had been suffering for ten years from inability to breathe through the right nostril, from which, also, there had been a constant flow of purulent matter during the same length of time. Anterior rhinoscopy showed that the mucous membrane of the inferior turbinated bone was so much swollen that it was impossible to see beyond its anterior extremity. On using the sound, a hard, uneven body was felt, which, with a little manipulation, was brought into view. This body was removed by forceps, piecemeal, without much pain. The centre of it was a metal button, on which there was deposited carbonate and phosphate of lime, together with organic substances. The patient, after the operation, complained that too much air passed through the nostril that had been affected, and that the throat soon felt dry; and on comparing the sizes of the inferior turbinated bones of the two nostrils, that of the affected side was much smaller, and so the nostril was larger than the other.

About 40 such cases have been published, and in the large majority some foreign body has formed a nucleus, on which deposit of salts has taken place.—*Deutsche Med. Zeitung*, Nov. 5th, 1885.

### Treatment of Whooping Cough by Resorcin.

Dr. Moncorvo, believing that pertussis is caused by parasitic agency, has tried painting the glottis with a 1 to 2 per cent. solution of chemically pure resorcin, and is much pleased with the result; but, as he remarks, the application with the brush is often resisted by the patient. (Anyone who has worked much with the laryngoscope amongst adults, and who knows the difficulty which is often met with in accurately painting the glottis, especially where there is a tendency to spasmodic cough, will appreciate this remark, especially seeing that the majority of patients who suffer from whooping cough are children.—Trans.)—*Deutsche Med. Zeit.*, Oct. 29th, 1885.

### The Use of Cocaine in Whooping Cough.

Dr. L. Barbillion, starting with the idea that the paroxysms of whooping cough are produced by irritation of the throat, and knowing, as we do, that cocaine is a very powerful anæsthetic for mucous membranes, has used the drug, with the following results: (1) A lessening of the number of attacks of coughing per diem. (2) Prevention of vomiting during the paroxysm, and of the debility produced thereby. (3) A greater power of resistance against the evil effects of a long and debilitating illness. No ill effects have been noted.

Seven cases are published *in extenso*, of which there were five cures: two cases were fatal, from broncho-pneumonia complicating the pertussis; one of these had measles also.

The drug was used in the form of a 5 per cent. solution in water, and the posterior pharyngeal wall was painted two to four times daily. (The cost of cocaine being now only about 4/- a gramme, the treatment has no disadvantage in point of costliness, and is certainly logical, and worthy of a trial.—Trans.)

### Contribution to the Question of a Mechanical Treatment of Whooping Cough.

Dr. Goldschmidt puts forth the hypothesis that in pertussis we have to do with an affection of the nose and the naso-pharyngeal cavity, and that the paroxysms of coughing are due to mucus entering the larynx, as the result of aspiration, or simply of gravity. The treatment to be adopted is to use an antiseptic nasal wash every two hours; and the author says that he has had good results.—*Rev. Mensuelle Laryngologie*, &c., October, 1885.

### On the Treatment of Catarrhal Phthisis, of Hæmoptysis, and of Chronic Bronchitis, by Terpine.

Germain Sée gives the following *résumé* of his paper on the "Treatment of Catarrhal Phthisis, of Hæmoptysis, and of Chronic Bronchitis, by Terpine:"

1. It diminishes and quickly arrests purulent expectoration in catarrhal forms of phthisis. Whether the muco-purulent secretions proceed from the bronchi, irritated by tubercles, or from the walls of pulmonary cavities; whether the malady is at an early stage, or at a phase of purulent breaking down, or even of cavities already formed; terpine should be used whenever the formation of pus is sufficiently abundant to tire the patient, to exhaust the strength, or to cause him to waste away.

2. It should be used with success in the hæmoptysis of the early stages of tuberculosis; that is to say, when the disease has not yet developed large cavities, with aneurisms of the pulmonary arteries.

3. In the treatment of pulmonary catarrhs; of chronic bronchitis not dependent on asthma, and only producing dyspnœa by choking the bronchi, terpine constitutes the best method of lessening bronchial hypersecretion.

4. The action is quick, sure, and free from physiological inconveniences, rendering it preferable to preparations of syrups of turpentine or tar, or of shoots of pine, which contain so little of it; and to essence of turpentine, which is not tolerated. It even offers advantages over creosote, on account of its perfect innocuity and easy digestion.

5. The best way of administering this medicine is either in the form of pills or tincture, and the best dose is one gramme.

6. In catarrhal, or emphysematous, or nervous asthma, which is to be distinguished from primary catarrh, iodine and pyridine have an incontestable superiority.—*Bulletin de l'Académie de Médicine*, No. 30, 1885.

### On the Treatment of Obesity.

Germain Sée, on "The Treatment of Obesity:"

1. A physiological regimen contains from 120 to 130 grammes of nitrogenous matter, obtained from 250 to 300 grammes of muscular flesh, or albuminates; of 100 to 120 gr. of neutral fats, plus 250 gr. of carbo-hydrates, supplied by 300 to 400 grammes of starch or sugar. These proportions should be modified so that the musculo-albuminous substances do not materially surpass the normal quantity; for meat taken in excess would of itself make fat. Fatty matters, easy of digestion, could, without inconvenience, be given in quantity amounting to from 60 to 90 gr.; the carbo-hydrates should be reduced to a minimum; as to vegetable food, it contains nothing nutritive.

2. Fluids, far from being suppressed, should be increased, to facilitate stomach digestion, and encourage general nutrition; but alcoholic drinks should be suppressed, especially ale; also mineral waters, as a rule: these should all be replaced by coffee and infusion of tea (as warm as possible).

3. Muscular exercises of all kinds are necessary to the obese; riding, which is a passive exercise, being excepted.

4. Vapour baths, warm baths, and, above all, hydropathy, offer some advantages.

5. Amongst medicines, the most useful are very small doses of iodides; chloride of sodium waters act only temporarily. Alkaline preparations and waters, especially good for fat diabetics, have no precise action on common obesity. All other medication is, to say the least, useless.—*Bulletin de l'Académie de Médicine*, No. 40, 1885.

**Cholera.**

The following are the conclusions on "Cholera," presented to the French Academy of Medicine by its Commission:

1. In the regions of France from which we have received answers from medical men, the cholera, as a rule, has only appeared as coming from a country already contaminated. For, in three-quarters of cases, this importation has been recognised as such; and for the other quarter, the importation is more than probable, for reasons set forth in the report.

2. If we trust entirely to observations contained in these reports, we see that cholera developes with less intensity in populous centres than in small localities. It is, therefore, a grievous error that the inhabitants of towns should, in time of epidemics, fly to the country.

3. A general want of cleanliness, and, above all, the bad habit of throwing human evacuations anywhere, is the principal cause of the propagation of the disease. For, in time of cholera, the dejections of a patient presenting only slight diarrhœa may contain cholera germs of a serious nature.

4. The germs of cholera are often spread by water contaminated by dejections of a patient, and it is generally in drinking this water that the disease is contracted.

5. The storms, that so often precede or aggravate cholera epidemics, act in contaminating the water by disseminating into them any impurities that may be lying about the soil.

6. It is because drinking waters are ordinarily well trapped and preserved from contaminating matter, that towns give more resistance to the spread of cholera. Nevertheless, a few towns supplied by river water lose this privilege.

7. In all cholera-haunted localities, however, low-lying districts situated near rivers, and places where the purity of the water is doubtful, are the most dangerous to inhabit.

8. Houses inhabited by cholera patients, as well as all dejections, clothes, or any other objects, should be thoroughly disinfected, according to the method prescribed by the Consulting Committee of Hygiene. Such precautions seem to have often arrested the spread of cholera at its beginning. But, to be entirely efficacious, medical men should exert great vigilance in prescribing disinfection; for the ignoring of early cases of cholera, or even of slight choleraic affections, may give rise to contamination of water, and propagation of the disease.

9. The chances of contracting cholera are greatly increased by old age, exhaustion, infancy (early), as well as by alcoholism and by general and personal want of cleanliness.

10. A first attack of cholera offers no immunity, even for a short time, against a second attack, inasmuch as numerous relapses have occurred even during a short epidemic.—*Bulletin de l'Académie de Médicine*, No. 35, 1885.

---

### Obituary.

